# Death from early colorectal cancer is predicted by the presence of transcripts of the REG gene family

**DOI:** 10.1054/bjoc.2000.1227

**Published:** 2000-06-15

**Authors:** R C A Macadam, A I Sarela, S M Farmery, P A Robinson, A F Markham, P J Guillou

**Affiliations:** Professorial Surgical Unit, University of Leeds, St James's University Hospital, Leeds, LS9 7TF, UK; Molecular Medicine Unit, University of Leeds, St James's University Hospital, Leeds, LS9 7TF, UK

**Keywords:** REG gene, colorectal cancer, regeneration, apoptosis

## Abstract

An intrinsic component of colorectal carcinogenesis may be the capacity to activate regenerative responses simultaneously with inhibition of apoptosis. Since apoptosis is known to be inhibited in colorectal cancer, this study sought evidence for the activation of the REG family of genes which are considered to be activated during regeneration of intestinal mucosa. Transcripts for the REG gene were found in 53% of colorectal cancers and for the PAP gene in 60% of colorectal cancers, by RT-PCR. Using in situ hybridization, the REG transcripts were found to be present in the tumour cells themselves rather than inflammatory or stromal cells. There were no significant correlations between the expression of these two genes and tumour stage, age or sex of the patient population or tumour site. However, in patients with non-metastatic disease who underwent ostensibly curative surgery, the expression of REG alone and co-expression of REG with PAP had a highly significantly adverse effect on survival. These data provide support for the concept that, in some tumours, carcinogenesis involves a regenerative process which co-exists with apoptotic inhibition and may provide a valuable selective indicator of the need for adjuvant therapy in those patients with early-stage colorectal cancer whose disease is destined to recur after curative surgery. © 2000 Cancer Research Campaign


*British Journal of Cancer* (2000) **83**(2), 188–195
© 2000 Cancer Research Campaign
doi: 10.1054/ bjoc.2000.1227, available online at http://www.idealibrary.com on IDEAL®

### Death from early colorectal cancer is predicted by the presence of transcripts of the REG gene family

**RCA Macadam[1], AI Sarela[1], SM Farmery[1], PA Robinson[2], AF Markham[2] and PJ Guillou[1]**

[1]Professorial Surgical Unit and [2]Molecular Medicine Unit, University of Leeds, St James's University Hospital, Leeds LS9 7TF, UK

**Summary** An intrinsic component of colorectal carcinogenesis may be the capacity to activate regenerative responses simultaneously with inhibition of apoptosis. Since apoptosis is known to be inhibited in colorectal cancer, this study sought evidence for the activation of the REG family of genes which are considered to be activated during regeneration of intestinal mucosa. Transcripts for the REG gene were found in 53% of colorectal cancers and for the PAP gene in 60% of colorectal cancers, by RT-PCR. Using in situ hybridization, the REG transcripts were found to be present in the tumour cells themselves rather than inflammatory or stromal cells. There were no significant correlations between the expression of these two genes and tumour stage, age or sex of the patient population or tumour site. However, in patients with non-metastatic disease who underwent ostensibly curative surgery, the expression of REG alone and co-expression of REG with PAP had a highly significantly adverse effect on survival. These data provide support for the concept that, in some tumours, carcinogenesis involves a regenerative process which co-exists with apoptotic inhibition and may provide a valuable selective indicator of the need for adjuvant therapy in those patients with early-stage colorectal cancer whose disease is destined to recur after curative surgery. © 2000 Cancer Research Campaign

**Keywords**: REG gene; colorectal cancer; regeneration; apoptosis

Colorectal cancer results from a series of genetic changes which disrupt the mechanisms that normally control growth in colonic epithelial cells (Fearon and Vogelstein, 1990; Kinzler and Vogelstein, 1996). There is considerable epidemiological and experimental evidence to suggest that an important environmental factor, implicated in the genetic mutations involved in sporadic colorectal tumorigenesis, is diet (Giovannucci and Willett, 1994) but the precise nature of the mutagens present in the diet is unclear (Bruce, 1987) and their relationship with the known genetic factors is undetermined. Kinzler and Vogelstein (1996) have suggested that mutagens may be carcinogenic not just because they induce mutation but also because they induce substantial cellular death with the corresponding induction of a regenerative response (Cohen and Ellwein, 1990). They hypothesized that this regenerative response, during which apoptotic signals must be turned off to enable net cellular growth to occur, would enable cells possessing mutations in growth-controlling genes to proliferate, whereas non-regenerating cells possessing these mutations would simply undergo programmed cell death. Although it is recognized that inhibitors of apoptosis such as BCL-2 are increased in colorectal cancer cells (Sinicrope et al, 1995) and that the induction of apoptosis by bile acids is diminished with bile from colorectal cancer patients (Garewal et al, 1996), evidence that genes directly involved in regenerative processes are sometimes activated in sporadic colorectal cancer is sparse.

The pancreatic regenerating gene (REG) is one member of a family of genes encoding at least three proteins, described in rats, mice and humans, which bear sequence homology to calcium-dependent lectins (Unno et al, 1993). The 166 amino-acid human REG gene product is known as Lithostathine and this is secreted by normal pancreatic acinar cells, but not islet cells, and belongs to the C-type lectin family. Another member of this family is known as pancreatitis-associated protein (PAP), the gene for which appears to be identical with the HIP gene (expressed in hepatocellular, intestine and pancreas carcinoma) and codes for a 175 amino-acid protein exhibiting 49% amino-acid identity with REG protein (Miyashita et al, 1995). These proteins have wide ranging functions in cellular adhesion and cell-to-cell interactions. The REG gene was initially identified during the screening of a cDNA library constructed from regenerating pancreatic tissue in a rat which had undergone partial pancreatectomy (Terazono et al, 1988). The human homologue of the rat reg gene is expressed in normal human pancreas (Bartoli et al, 1993), was sequenced in 1990 (Watanabe et al, 1990), and has been localized to chromosome 2p12 (Lasserre et al, 1994). A preliminary tissue distribution study using northern blot analysis suggested that REG gene expression might occur in six of seven colorectal cancers, but not in normal colonic mucosa from the same patients (Watanabe et al, 1990). Members of this gene family are up-regulated in human colorectal cancer cell lines during differentiation (Bernard-Perrone et al, 1999), this being reflected at the protein level by western blotting studies in a small series of human colorectal cancers (Rechreche et al, 1999). REG gene expression has also been implicated in the regenerative process in gastric mucosa (Asahara et al, 1996) and its product has been detected in ductal adenocarcinoma of the pancreas (Kimura et al, 1992). PAP is expressed in 25% of human hepatocellular carcinomas and has been implicated in liver cell proliferation and differentiation (Lasserre et al, 1992).

Because of the potential importance of the REG gene family products in cellular adhesion and regenerative processes, the

*Received 14 October 1999*
*Revised 18 February 2000*
*Accepted 24 February 2000*

*Correspondence to:* PJ Guillou

**Table 1** REG/PAP positivity in relation to clinical parameters

| Variable | Patients (*n*) | REG-positive | PAP-positive | PAP and REG-positive |
|---|---|---|---|---|
| Total numbers studied (REG/PAP) | 142/130 | | | |
| Mean age (years ± S.D.) | 70±10 | 71±10 | 71±9 | 70±10 |
| Sex | | | | |
| Male | 83/142 (58.5%) | 47/83 (57%) | 40/71 (56%) | 24/71 (34%) |
| Female | 59/142 (41.5%) | 28/59 (47%) | 38/59 (64%) | 17/59 (29%) |
| Total | 142/130 | 75/142 (53%) | 78/130 (60%) | 41/130 (31.5%) |
| Tumour site | | | | |
| Colon | 87/142 (61%) | 42/87 (48%) | 43/75 (57%) | 21/75 (28%) |
| Rectum | 55/142 (39%) | 33/55 (60%) | 35/55 (64%) | 20/55 (36.4%) |
| Tumour stage | | | | |
| I ($T_{1–2}N_0M_0$) | 18 | 12/18 (67%) | 14/18 (77%) | 7/18 (39%) |
| II ($T_{3–4}N_0M_0$) | 60 | 32/60 (53%) | 31/55 (56%) | 16/55 (29%) |
| III ($T_{1–4}N_{1–2}M_0$) | 46 | 23/46 (50%) | 26/42 (62%) | 16/42 (38%) |
| IV ($T_{1–4}N_{0–2}M_1$) | 18 | 8/18 (44%) | 7/15 (47%) | 2/15 (13%) |

present study aimed to determine the precise frequency at which these genes are expressed in a series of colorectal cancers. We also aimed to determine the relationship between transcription of the REG family of genes and clinical outcome in patients with colorectal cancer treated by surgical resection alone without adjuvant therapy.

### MATERIALS AND METHODS

### Cell lines and clinical material

Preliminary studies were undertaken to determine REG expression in a variety of human tumour cell lines of colorectal (LoVo, COLO320, SW480, SW948), pancreatic (PANC1), hepatic (hepG2) and gastric (KATO-III) origin. Heparinized peripheral venous blood was obtained from healthy volunteers and neutrophil and mononuclear cell fractions isolated by Dextran sedimentation and Ficoll extraction. Normal bone marrow samples were obtained from ten patients undergoing bone marrow aspiration for investigatory purposes unrelated to the presence of malignant disease. Samples of non-ulcerated tumour edge, taken from the bowel immediately following resection, were obtained from 142 patients undergoing surgery for colorectal cancer of whom 83 were male and 59 female. Their mean (±SD) age was 70 (±10) years. Fifty five of these patients suffered from rectal cancer, the remaining 87 having colonic cancer. The TNM staging of these tumours is shown in Table 1. Normal colonic mucosa from a point distant to the tumour was also available from 88 of the fresh surgically resected specimens. REG expression was determined in all 142 colorectal cancers but PAP expression was only examined in RNA preparations from 130 of these tumours because of paucity of material from the remaining twelve. At the time at which these specimens were collected, no patient with stage I or II disease received adjuvant chemotherapy and only five patients with stage III disease underwent adjuvant therapy with 5-fluorouracil and folinic acid.

Normal colonic mucosa was also obtained from eight patients undergoing colectomy for diverticular disease. A sample of fresh normal pancreas was obtained from the resection specimen of a young patient undergoing distal pancreatectomy for blunt trauma to the pancreas. Fresh polyp tissue was obtained from three patients undergoing colonoscopic resection of adenomatous polyps. In addition, 20 colonic mucosal biopsies were obtained from 15 patients suffering from ulcerative colitis during an active phase of their disease and 16 mucosal biopsies were also provided by these same patients between 1 and 6 months after their disease had been in remission subsequent to appropriate therapy.

All specimens for RNA assay were snap-frozen in liquid nitrogen and stored at –70°C until assay. The presence of tumour in the malignant tissues (or absence of tumour in the ‘normal’ tissue) was confirmed histologically in all samples. The remaining operative specimen was sent for routine histological examination and pathological staging.

Clinical follow-up of up to 5 years duration (median 24 months, mean 33 months, range 6–60 months) was conducted on all patients, and in particular the clinical outcome in relation to REG and PAP status was determined in all patients who underwent resection which was considered to be potentially curative. Patients who died of non-cancer-related causes were treated as censored observations in the disease-specific survival analysis.

### RNA extraction

Small samples (<10 mg) of mucosa and tumour were taken from frozen tissue blocks, ground to powder in liquid nitrogen and immediately suspended in 1 ml of Catrimox™ cationic surfactant solution (Iowa biotechnology Corp. USA). The neutrophils, monocytes, bone marrow samples and tumour cell lines were similarly treated. Particulate remains were allowed to settle for 5 min and the supernatants aspirated to a fresh tube for further processing. The supernatant was centrifuged for 5 min at 1000 ***g***, forming a detergent-bound RNA pellet. The absence of contaminating genomic DNA was confirmed by PCR using genomic primers (Heatshock protein 70, accession number M11717 primers at 281…304 and 485…462 bp in the coding sequence, respectively).

### Reverse transcriptase-polymerase chain reaction (RT-PCR).

RNA (10 μl of the above solution) was reverse transcribed and cDNA samples underwent PCR amplification using primers for a

© 2000 Cancer Research Campaign constitutively expressed gene (glyceraldehyde-3-phosphate dehydrogenase), to confirm the reproducibility of mRNA extraction and reverse transcription. Intron-spanning primers were devised to amplify a 283 base-pair product of REG cDNA (accession number J054 12) (REG upper dAACATGAATTCGGGCAACC, positions 2707…2726, exon 4; REG lower dAGGAGAACTTGTCTTCACAA, positions 3900…3881, exon 6). Similarly, to detect PAP (accession number L15533) transcripts, primers with the following sequences were employed: PAP upper dTGTCACCAAAATCCTGGACA, positions 2060…2079, exon 3; PAP lower dGGATTTCTCTCCCATGCAAA, positions 3097…3116, exon 5. Primers (20 pmol) and template (1 μl of RT reaction product) in a final volume of 10 μl were overlaid with mineral oil and heated to 95°C for 'hot start' PCR. The reaction was initiated by the addition of 10 μl of a PCR reaction mixture, to give Taq DNA polymerase (1 U), dNTPs (0.2 mM each, Pharmacia), 1 × Taq buffer (Promega) and $MgCl_2$ (2 mM). Reactions proceeded in a thermal cycler for 40 cycles (denaturation 60 s 95°C, annealing 60 s 55°C, extension 60 s 72°C). PCR products were electrophoresed on 2% agarose gels containing ethidium bromide and visualized by ultraviolet transillumination. REG positivity was defined as the presence of a 283 bp band and PAP as a 244 bp band in each of three independent reactions and the presence of the control 177 bp glyceraldehyde-3-phosphate dehydrogenase RT-PCR product. Confirmation that the observed product produced by the above PCR was derived from the amplification of REG 1A or PAP cDNA was confirmed by direct sequencing using the Sequenase version 2.0 DNA sequencing kit (Amersham, Little Chalfont, Buckinghamshire, UK).

### In situ hybridization studies

Because previous tissue localization studies for PAP protein in the digestive tract had shown no expression in normal colonic mucosa (Masciotra et al, 1995), in situ hybridization studies for REG mRNA only was performed. The in situ hybridization was carried out using the Boehringer Mannheim (Lowes, East Sussex, UK) protocol and reagents on 4 micrometer-thick microtome sectioned OCT-embedded snap-frozen tissue samples.

#### *PCR amplification and probe labelling*

Primers incorporating T7 and T3 promoter sequences were synthesized to amplify REG gene-specific sequences (REG T7 dATGCTAATACGACTCACTATAGGGTGC CAGAACATGAATTC, REG dAGGAGAACTTGTCTTCACAA) (REG T3 dATGCTTAACCCTCACTAAAGGGAATTGACTTGCAGACAAA, REG dAACATGAATTCGGGC). PCR conditions were the same for both primer pairs and were as described above under PCR methodology. The dioxygenin (DIG) labelling reaction contained 100–200 ng of purified PCR product, 2 μl of 10 × concentrated DIG RNA labelling mix, 2 μl of 10 × concentrated transcription buffer, 2 μl RNA polymerase (T7 or T3) and water to a reaction volume of 20 μl. Estimation of RNA probe yield was carried out in a side-by-side comparison of the DIG-labelled sample nucleic acid from each PCR amplification with the DIG-labelled control provided in the kit.

#### *In situ hybridization*

Air-dried sections were treated with PBS containing 0.3% Triton X-100 (Sigma), permeabilized for 30 min at 37°C with TE buffer containing 1 μg $ml^{-1}$ RNase-free Proteinase K (Boehringer Mannheim). Sections were then fixed with PBS containing 4% paraformaldehyde, and were then acetylated with 0.25% (v/v) acetic anhydride (Sigma). Separate solutions were made of REG sense and antisense DIG-labelled RNA in hybridization buffer, and 10 ng in 30 μl of solution was added to the sections which were then incubated at 42°C overnight in a humid chamber. In order to digest unbound probe, sections were incubated at 37°C for 30 min in NTE buffer containing 20 μg $ml^{-1}$ RNase A. The sections were then incubated for 2 h with a 1:500 concentration of sheep anti-DIG-alkaline phosphatase (Fab fragment). A colour solution was prepared containing nitroblue tetrazolium, 5-bromo-4-chloro-indolyl phosphate with 1 mM levamisole, and 200 μl of this solution applied to each slide which was then incubated in a humidified chamber in the dark overnight, prior to staining with 0.1% nuclear-fast red. Adjacent sections did not undergo in situ hybridization but were stained with haematoxylin and eosin to confirm the histological features of the tissue under investigation.

Positive control samples for REG expression consisted of normal, freshly resected human pancreas and samples of colorectal cancer demonstrated to express REG on RT-PCR examination. In situ hybridization for REG was performed on sections of tumours which had been found to express on RT-PCR, as well as tumours found to be negative for REG on RT-PCR, and samples of normal colonic mucosa. Negative controls were provided by attempting hybridization with the sense probe and by omitting any probe from the hybridization solution.

### Statistical analysis

Incidence data for REG and PAP gene transcription positivity amongst the colorectal tumours and other tissues was analysed by Chi-squared analysis. Kaplan-Meier survival plots were generated for patients with or without tumour REG and/or PAP expression and for co-expressed gene expression, and the significance of any observed differences was determined by log-rank analysis. Cox regression analysis was employed to determine the contribution of clinico-pathological factors and REG and PAP mRNA expression towards patient survival and disease-free interval after surgical resection. Entry into the model was via the forward stepwise (Likelihood ratio) method and overall survival was defined as the time from surgery to the time of death. Endpoints were censored for patients dying of causes other than metastatic colorectal cancer. All $P$ values for these analyses are two-sided.

### RESULTS

### REG and PAP expression in cell lines, neutrophils, mononuclear cells and normal bone marrow

The colon adenocarcinoma cell line SW948 was found to express REG and PAP mRNA and was utilized throughout as a positive control. Other colorectal (LoVo, COLO320, SW480), pancreatic (PANC1), hepatic (hepG2) and gastric (KATO-III) tumour cell lines did not express either REG or PAP (Figure 1). Furthermore, no specific products were obtained with PCR amplification of cDNA from healthy peripheral blood neutrophils, mononuclear cells, normal bone-marrow cells or from human total genomic DNA. Both REG and PAP were expressed in normal pancreas.

 © 2000 Cancer Research Campaign

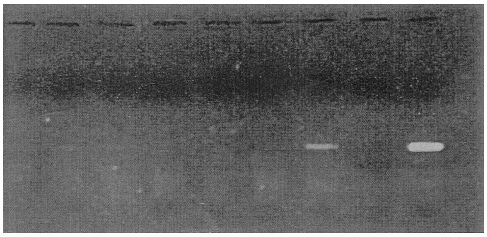

**Figure 1** REG RT-PCR was performed on template cDNA derived from eight cultured cell lines. mRNA was extracted from the cultured cells and reverse transcribed. cDNA integrity was confirmed in each sample by the successful amplification of G3PDH (not shown). PCR was conducted with oligonucleotide primers specific for REG and the products resolved by agarose electophoresis as shown above where amplification of REG cDNA yielded a 283 bp PCR product. No REG expression was detected in LoVo (Lane 1), Colo320 (Lane 2), SW480 (Lane 3), PANC1 (Lane 4), HepG2 (Lane 5) or KATOIII (Lane 6). REG cDNA was detected only in SW948 (Lane 7). A template of water only was included as a negative control (Lane 8). Template cDNA derived from a sporadic colon carcinoma known to express REG and PAP was included as a positive control (Lane 9). Identical results were obtained from PCR in which oligonucleotide primers specific for PAP were utilized (data not shown). Separate RT-PCR reactions demonstrated that REG cDNA was not present in cDNA derived from normal neutrophils, monocytes or from samples of normal bone marrow (not shown).

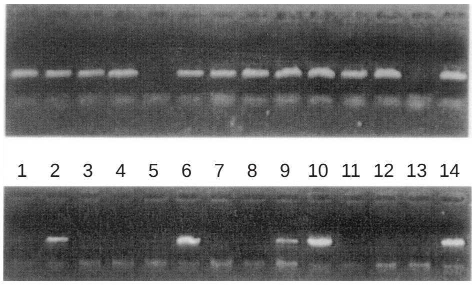

**Figure 2** G3DPH and REG PCR on paired samples of normal colorectal mucosa and primary adenocarcinoma from six patients undergoing colorectal cancer resection. G3PDH PCR confirmed the integrity of reverse transcription in 11 of 12 samples. Failure of reverse transcription in sample 5 invalidated the result of the REG PCR and repeat RNA extraction and RT-PCR were subsequently performed on this sample. Lanes 1 and 2 Patient 1, Normal tissue and cancer tissue; Lanes 3 and 4 Patient 2, Normal tissue and cancer tissue; Lanes 5 and 6 Patient 3, Normal (Void) and cancer tissue; Lanes 7 and 8 Patient 4, Normal tissue and cancer tissue; Lanes 9 and 10 Patient 5, Normal tissue and cancer tissue; Lanes 11 and 12 Patient 6, Normal tissue and cancer tissue; Lane 13, Water negative control; Lane 14, SW948 positive control.

### REG expression in human colorectal tissues

Analysis of the 142 primary colorectal adenocarcinoma samples (Table 1) demonstrated that 75 of 142 tumours (53%) expressed REG mRNA. In contrast, REG mRNA expression was detected in only 16 of the 88 (18.1%) available normal mucosa specimens from the same patients ($P = 0.02$). In all but two cases the expression in normal colonic mucosa occurred only when adjacent tumour samples from the same patients also expressed REG. (Figure 2). The incidence of REG positivity was higher in tumours of rectal rather than colonic origin (60% vs 48%), but this was not statistically significant. Within the colon, REG expression was almost equally distributed between left- (49%) and right- (48%) sided lesions. Thus, overall analysis of REG mRNA expression by histological stage revealed a similar incidence of positivity at all stages of the disease with no statistically significant association with any particular age, gender, site of tumour origin or tumour stage (Table 1).

Positive amplification of REG transcripts was obtained in only one of the eight samples of colonic mucosa from the patients undergoing colectomy for diverticular disease. None of the colonic polyps were REG-positive. Of the colonic biopsies obtained from patients with active ulcerative colitis, 11 of 20 were positive for REG mRNA compared with three of 16 biopsies obtained from the same patients when the disease was in remission and histologically normal (Chi-squared = 4.91, $P < 0.05$).

### PAP expression in human colorectal tissues.

Determination of PAP expression in colorectal tissues was technically successful in 130 of the 142 tumours studied for REG expression. PAP transcripts were identified in 78 of these tumour specimens and in 31 of 72 corresponding normal colorectal mucosa specimens. Again, PAP positivity in normal mucosa was only seen in samples of mucosa obtained from colons bearing PAP -positive tumours and not in colonic mucosa from non-tumour-bearing colons. There were no statistically significant correlations between tumour PAP -positivity and age, sex, tumour site or stage. In a significant proportion of tumours (32% overall) both PAP and REG were co-expressed but again this had no significant correlation with tumour stage, sex, age or site (Table 1). There was insufficient material available to determine the PAP status in the colitic biopsies.

### Correlation between tumour REG and PAP expression and survival

Multivariate analysis of the whole group of colorectal cancer patients revealed stage to be a significant independent determinant of survival, as expected when death from colorectal cancer is the event of interest and survival time is the variable (Chi-squared = $P < 0.00001$). Overall, REG expression failed to emerge as a statistically significant independent variable for the whole group when analysed in relation to survival time and death from colorectal cancer, irrespective of whether the stage IV cases were included or not. Kaplan–Meier curves were constructed for each of the different pathological stages according to REG and/or PAP positivity and this revealed that in the stage III and IV groups, neither PAP nor REG positivity had any significant impact on survival. For all patients in stages I, II and III who underwent surgical resection with curative intent, REG positivity had a negative impact on disease-free survival (Figure 3) as did PAP positivity in the node-negative group, but in neither instance did this reach statistical significance on log-rank testing. However, in the group of 78 patients with no metastases either clinically or histologically, the expression of REG had a deleterious effect on survival on log-rank testing ($P = 0.0307$) (Figure 4). Similarly, although in the total patient group overall the expression of REG had no impact on disease-free interval, in the 78 patients with stages I and II ($T_{1–2}$, $N_0M_0$) disease undergoing potentially curative resection, REG positivity was the only variable in the Cox proportional hazards model capable of independently predicting tumour recurrence ($P < 0.01$) with a relative risk of 4.25 (95% CI 1.008–17.81). PAP expression had a similarly deleterious effect on survival in the node-negative group but this was not statistically significant. However, when the results of REG and PAP co-expression were

*© 2000 Cancer Research Campaign*

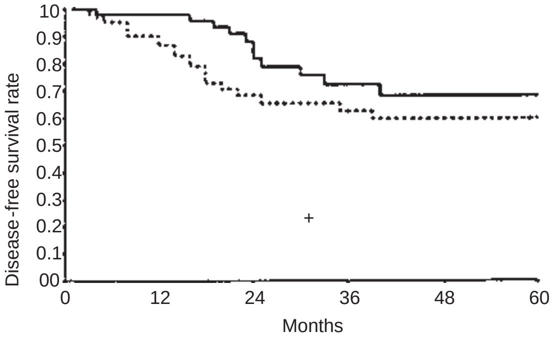

Number at risk

| | | | | | | |
|---|---|---|---|---|---|---|
| 56 | 46 | 26 | 16 | 16 | 14 | REG– |
| 68 | 48 | 23 | 21 | 19 | 14 | REG+ |

**Figure 3** Disease-free survival of patients with stages I, II and III colorectal cancer. The broken line represents patients with tumours that expressed REG mRNA whereas the solid line represents patients with tumours that did not express REG.

Number at risk

| | | | | | | |
|---|---|---|---|---|---|---|
| 34 | 26 | 18 | 11 | 10 | 10 | REG– |
| 44 | 28 | 12 | 10 | 8 | 5 | REG+ |

**Figure 4** Disease-free survival of patients with stages I and II carcinoma of the colon and rectum. Again the broken line represents patients with tumours that expressed REG mRNA whereas the solid line represents patients with tumours that did not express REG.

Number at risk

| | | | | | | |
|---|---|---|---|---|---|---|
| 22 | 21 | 13 | 8 | 8 | 8 | REG– / PAP+ |
| 11 | 9 | 6 | 3 | 3 | 3 | REG– / PAP– |
| 16 | 14 | 8 | 6 | 4 | 4 | REG+ / PAP– |
| 23 | 16 | 7 | 6 | 5 | 3 | REG+ / PAP+ |

**Figure 5** The expression of PAP mRNA by stages I and II tumours in addition to REG mRNA distinguishes a population of patients with histologically early stage disease who have a poor prognosis.

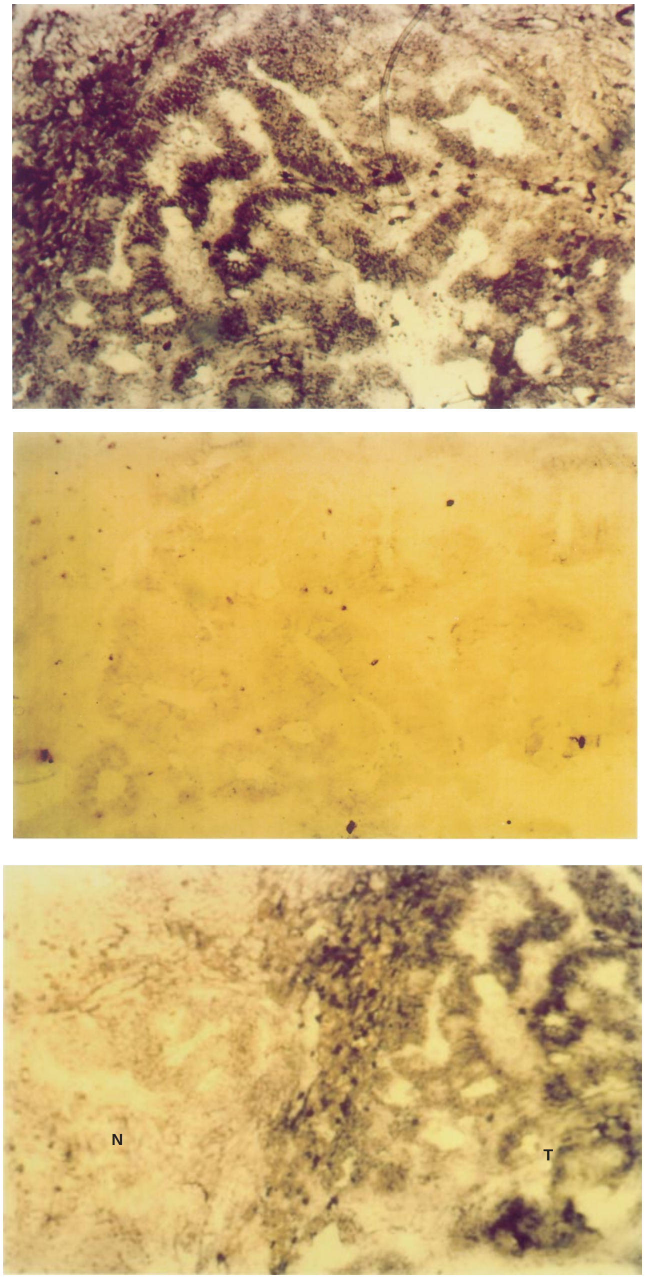

**Figure 6** (**A**) In situ hybridization using REG antisense riboprobe demonstrating strong staining of the epithelial cell component of a well-differentiated colon adenocarcinoma, indicating the presence of REG mRNA (× 100). (**B**) In situ hybridization using REG sense probe on a section from the same tissue sample as in **A** demonstrating no specific binding of this riboprobe to the malignant epithelium (× 100). (**C**) In situ hybridization using REG antisense probe demonstrating hybridization of the riboprobe with RNA within tumour cells at the interface between tumour (T) and normal mucosa. Minimal non-specific binding was observed with non-neoplastic colorectal epithelium (× 100).

examined, there was a significant trend indicating a diminished 5-year survival in node-negative patients whose tumours co-expressed REG and PAP (Figure 5) (Chi-squared = 8.46, $P$ = 0.037). There were insufficient patents with stage III disease who received adjuvant chemotherapy to determine whether or not expression of these genes had a relationship to survival with or without adjuvant therapy.

*© 2000 Cancer Research Campaign*

### In situ hybridization studies

Sections of normal pancreas elicited the expected strong reaction in acinar cells, but not in pancreatic islets, on in situ hybridization with the antisense probe, but not the sense probe. Tumours found to be REG-positive by RT-PCR elicited a strongly positive reaction on in situ hybridization with the REG antisense probes. The distribution of the hybridization product was within the epithelial-derived tumour cells rather than the stromal or infiltrating inflammatory cells (Figure 6A, and 6C). Sections incubated with negative control REG sense riboprobe failed to elicit a positive reaction (Figure 6B). None of the RT-PCR REG-negative tumours elicited a positive reaction with either the antisense or the sense probes.

### DISCUSSION

The initial purpose of this investigation was to seek evidence in support of the hypothesis that regenerative processes are activated during colorectal tumorigenesis (Kinzler and Vogelstein, 1996). The REG gene is known to be expressed in the rat pancreas following 90% pancreatectomy and it has been demonstrated that REG is induced by the action of several mitogens known to induce islet cell proliferation (Watanabe et al, 1994). The present data show that transcription of the REG family of proteins (REG and PAP) is a feature of many human colorectal adenocarcinomas. In these tumours REG expression was independent of tumour stage or site although there was a tendency for it to be more frequently expressed in rectal cancers than those originating in the colon. The presence of REG or PAP mRNA in normal mucosa from the colorectal cancer patients was almost invariably associated with the presence of a REG-positive tumour and could be explained by contamination with viable tumour cells shed from the tumour, since these ‘normal’ samples were always removed from macroscopically normal mucosa at a longitudinal resection margin. The explanation for the presence of REG transcripts in the ‘normal’ mucosa from the one patient with diverticular disease and the two surgical specimens apparently containing REG-negative tumours is less obvious (see below). PAP and its protein are already known to be present in human Paneth cells and goblet cells of the small bowel but are undetectable in the large bowel mucosa (Masciotra et al, 1995).

Without the data from the in situ hybridization experiments, it could be argued that the frequency with which REG expression is seen during the acute phases of ulcerative colitis (an inflammatory bowel disease known to carry a significant long-term risk of the development of dysplasia and carcinoma) suggests that it is the inflammatory component which is the source of REG expression in tumours. In histologically normal, quiescent colitic mucosa the expression of REG subsided, again in keeping with an origin in the inflammatory cell infiltrate. However, the in situ hybridization experiments appear to exclude stromal and inflammatory cells as the source of the REG mRNA, this being confined to the epithelial component of the malignant tumour, with no positive reaction being observed in the adjacent normal colonic mucosa or in RT-PCR-negative tumours. The use of REG sense probes confirmed the specificity of the REG antisense probes for this in situ reaction. This was reinforced by the finding that pancreatic acinar cells also provided a positive reaction with the antisense probe but not with the sense probe as expected (Kimura et al, 1992).

The waxing and waning of REG expression with ulcerative colitis disease activity may reflect activation of the epithelial regenerative process during healing of the mucosal injury, followed by down-regulation once healing is complete. It is recognized that other alterations of gene function, such as DNA mismatch repair, also occur in dysplastic and malignant epithelium in chronic ulcerative colitis (Suzuki et al, 1994). At this stage, data on the expression of REG in ulcerative colitis cannot contribute further to this debate because specimens of dysplastic colitic mucosa were not available during the present investigation. However, the fact that REG mRNA was detected in one colorectal cancer cell line but not in peripheral blood neutrophils, mononuclear cells (lymphocytes and monocytes) or normal bone-marrow cells supports the hypothesis that REG expression in colorectal cancer is not attributable to the inflammatory cell infiltrate often seen in these tumours but that it is of carcinomatous epithelial cell origin. Final confirmation of the translation of REG gene product by colorectal cancer cells awaits immunohistochemical studies for which monoclonal antibodies are currently in preparation. If the tumour distribution of the REG gene product is confirmed immunohistochemically then this would be consistent with a growth-promoting role for REG in colorectal carcinoma. There is evidence that the REG gene product may regulate rat pancreatic islet regeneration by means of a direct trophic effect on the islet cells or their precursors (Watanabe et al, 1994; Zenilman et al, 1996), and the protein has structural homology with the growth-promoting lectins (Lasserre et al, 1994; Patty, 1988). Moreover, recent data suggest that rodent REG gene expression is actually inhibited during cellular differentiation (Zenilman et al, 1997). These activities are entirely compatible with the notion that during colorectal carcinogenesis, this regenerative gene is activated during the repair response associated with mutagen-induced colonic epithelial cell death as propounded by Kinzler and Vogelstein (1996). This regenerative response may switch off apoptotic signals, as is known to occur in colorectal carcinogenesis, (Bedi et al, 1995) and permit the survival of those cells with mutations in other growth-promoting or metastasis-inducing genes. Thus, those cells exhibiting both a regenerative response and other genetic mutations would have a survival advantage, whereas mutated cells devoid of a regenerative response would proceed down the apoptotic pathway. At present there is no direct evidence to indicate that the REG gene product can directly participate in apoptotic inhibition but it will be of interest to determine, for example, the relationship between REG expression and that of the recently-described inhibitor of apoptosis, survivin (Ambrosini et al, 1997). REG is a member of a family of at least three structurally-related genes, one of which (PAP) has been shown to be associated with hepatocellular carcinoma (Lasserre et al, 1994). It is of interest to find that PAP is also expressed in a proportion of colorectal cancers whereas it is not normally expressed in colonic mucosa. The mechanisms altering the transcriptional control of this family of genes may also be of interest from a therapeutic standpoint.

Few of the common molecular genetic alterations in colorectal cancer serve as prognostic indicators of survival (Dix et al, 1994*b*). The immunohistochemical detection of nuclear p53 over-expression has been variously reported as being associated with shorter survival (Auvinen et al, 1994; Zeng et al, 1994), longer survival (Dix et al, 1994*a*) or with no impact on survival (Scott et al, 1991), while cytoplasmic p53 over-expression has been correlated with shorter survival (Sun et al, 1992). In the proximal

© 2000 Cancer Research Campaign colon, microsatellite instability has been reported to be an indicator of good prognosis (Thibodeau et al, 1993), a paradox which seems difficult to explain especially since allelic imbalance for chromosome 8p appears to be associated with a survival advantage (Halling et al, 1999). There was a significant increase in colorectal cancer-related deaths in the sub-group of patients with stages I and II, non-metastatic early disease whose tumours expressed REG either alone or in combination with PAP. Furthermore, the co-expression of REG and PAP was the only variable capable of confidently identifying that group of patients with early stage disease who were destined to develop recurrence despite 'curative' surgery, although the confidence intervals for these data are wide and limit the statistical power of the analysis. However, if this observation is confirmed in a larger series then it will be of particular importance because of the potential to discern those patients with histologically non-metastatic disease who are at high risk of recurrence and who might benefit from the administration of adjuvant chemo- or radiotherapy. Such treatment is undoubtedly effective for patients with node-positive colon cancer (Moertel et al, 1995) in whom the recurrence rate is as high as 70% and for whom the potential survival advantages of chemotherapy outweigh the risks and costs of over-treating the remaining 30% who will not develop recurrent disease. Similar arguments surround the use of radiotherapy for rectal cancer (Minsky, 1997). However, the indiscriminate administration of such therapy to the 90% or 70% of patients with stages I and II colorectal cancer respectively who will not develop recurrent disease, is rather more controversial. In this group, it has become a major clinical goal to distinguish those patients who are at high risk of developing recurrent disease from the majority who will not, so as to avoid the costs and toxicity of adjuvant therapy in the latter and concentrate resources on the former. It is possible that the expression of REG and PAP and possibly other members of this gene family may provide the prognostic marker which identifies such high-risk patients and allows this therapeutic goal to be realised. However, RT-PCR is impractical for routine clinical use and we are currently examining the possibilities of immunohistochemistry in order to confirm these data and enable the clinical application of these findings.

In conclusion, this study provides evidence that the activation of a regenerative process plays an oncogenic role in a significant proportion of human colorectal cancers. The manifestation of this regenerative process in the form of transcription of the REG gene family permits the identification of a group of patients who are at high risk of developing recurrent colorectal cancer following 'curative surgery' for apparently non-metastasizing disease and who may potentially benefit from the administration of adjuvant therapy.

### ACKNOWLEDGEMENTS

We are grateful to the Medical Research Council, The Royal College of Surgeons of England and Zeneca Diagnostics for financial support in the conduct of this work.

### REFERENCES

Ambrosini G, Adida C and Altieri DC (1997) A novel anti-apoptosis gene, *survivin*, expressed in cancer and lymphoma. *Nat Med* **3**: 917–921

Asahara M, Mushiake S, Shimada S, Fukui H, Kinoshita Y, Kawanami C, Watanabe T, Tanaka S, Ichikawa A, Ichiyama Y, Narushima Y, Takasawa S, Okamato H and Chiba MTT (1996) *Reg* gene expression is increased in rat gastric enterochromaffin-like cells following water immersion stress. *Gastroenterology* **111**: 45–55

Auvinen A, Isola J, Visakorpi T, Koivula T, Virtanen S and Hakama M (1994) Overexpression of p53 and long-term survival in colorectal cancer. *Br J Cancer* **70**: 293–296

Bartoli C, Gharib B, Giorgi D, Sansonetti A, Dagorn J.-C and Berge-Lefranc J.-L (1993) A gene homologous to the *reg* gene is expressed in human pancreas. *FEBS Lett* **327**: 289–293

Bedi A, Pastricha PJ, Akhta AJ, Barber JP, Bedi GC, Giardiello FM, Zehnbauer BA, Hamilton SR and Jones RJ (1995) Inhibition of apoptosis during development of colorectal cancer. *Cancer Res* **55**: 1811–1816

Bernard-Perrone FR, Renaud WP, Guy-Crotte OM, Bernard P, Okamoto H, Balas DC and Senegas-Balas FO (1999) Expression of REG protein during cell growth and differentiation of two human colon carcinoma cell lines. *J Histochem Cytochem* **47**: 863–870

Bruce WR (1987) Recent hypotheses for the origin of colon cancer. *Cancer Res* **47**: 4237–4242

Cohen SM and Ellwein LB (1990) Cell proliferation in carcinogenesis. *Science* **249**: 1007–1011

Dix B, Robbins P, Carrello S, House A and Iacopetta B (1994*a*) Comparison of p53 gene mutation and protein overexpression in colorectal carcinomas. *Br J Cancer* **70**: 585–590

Dix BR, Robbins P, Soong R, Jenner D, House AK and Iacopetta BJ (1994*b*) The common molecular genetic alterations in Dukes' 'B' and 'C' colorectal carcinomas are not short-term prognostic indicators of survival. *Int J Cancer* **59**: 747–751

Fearon ER and Vogelstein B (1990) A genetic model for colorectal tumorigenesis. *Cell* **61**: 759–767

Garewal H, Bernstein H, Bernstein C, Sampliner R and Payne C (1996) Reduced bile acid apoptosis in 'normal' colorectal mucosa; a potential biological marker for cancer risk. *Cancer Res* **56**: 1480–1483

Giovannucci E and Willett WC (1994) Dietary factors and risk of colon cancer. *Ann Med* **26**: 443–452

Halling KC, French AJ, McDonnell SK, Burgart LJ, Schaid DJ, Peterson BJ, Moon-Tasson L, Mahoney MR, Sargent DJ, O'Connell MJ, Witzig TE, Farr GH, Goldberg RM and Thibodeau SN (1999) Microsatellite instability and 8p allelic imbalance in stage B2 and C colorectal cancers. *J Natl Cancer Inst* **91**: 1295–1303

Kimura N, Yonekura H, Okamoto H and Nagura H (1992) Expression of human regenerating gene mRNA and its product in normal and neoplastic human pancreas. *Cancer* **70**: 1857–1863

Kinzler KW and Vogelstein B (1996) Lessons from hereditary colorectal cancer. *Cell* **87**: 159–170

Lasserre C, Christa L, Simon MT, Vernier P and Brechot C (1992) A novel gene (HIP) activated in human primary liver cancer. *Cancer Res* **52**: 5089–5095

Lasserre C, Simon MT, Ichikawa H, Dirong S, Nguyen VC, Christa L, Vernier P and Brechot C (1994) Structural organization and chromosomal localization of a human gene (HIP/PAP) encoding a C-type lectin overexpressed in liver cancer. *Eur J Biochem* **224**: 29–38

Masciotra L, Porte PLDL, Frigierio J-M, Dusetti NJ, Dagorn J-C and Iovanna JL (1995) Immunocytochemical localization of pancreatitis-associated protein in human small intestine. *Dig Dis Sci* **40**: 519–524

Minsky BD (1997) Adjuvant therapy for rectal cancer – a good first step. *New Eng J Med* **336**: 1016–1017

Miyashita H, Nakagawara K, Mori M, Narushima Y, Noguchi N, Moriizumi S, Takasawa S, Yonekura H, Takeuchi T and Okamoto H (1995) Human REG family genes are tandemly ordered in a 95-kilobase region of chromosome 2p12. *FEBS Lett* **377**: 429–433

Moertel CG, Fleming TR and MacDonald JS (1995) Fluorouracil plus levamisole as effective therapy after resection of stage III colon carcinoma: a final report. *Ann Intern Med* **122**: 321–326

Patty L (1988) Homology of pancreatic stone protein with animal lectin. *Biochem J* **253**: 309–311

Rechreche H, Montalto G, Mallo GV, Vasseur S, Marasa L, Soubeyran P, Dagorn J and Iovanna JL (1999) *pap*, *reg*1a, and *reg*1b mRNAs are concomitantly up-regulated during human colorectal carcinogenesis. *Int J Cancer* **81**: 688–694

Scott N, Sagar P, Stewart J, Blair GE, Dixon MF and Quirke P (1991) p53 in colorectal cancer: clinicopathological correlation and prognostic significance. *Br J Cancer* **63**: 317–319

Sinicrope FA, Cleary SBRKR, Stephens LC, Lee JJ and Levin B (1995) *bcl*-2 and p53 oncogene expression during colorectal tumorigenesis. *Cancer Res* **55**: 237–241

 © 2000 Cancer Research Campaign

Sun X-F, Carstensen JM, Zhang H, Stal O, Wingren S, Hatschek T and Nordenskjold B (1992) Prognostic significance of cytoplasmic p53 oncoprotein in colorectal adenocarcinoma. *Lancet* **340**: 1369–1373

Suzuki H, Harpaz N, Tarpin L, Ying J, Jiang H-Y, Bell JD, Hontanosas M, Groisman GM, Abraham JM and Meltzer SJ (1994) Microsatellite instability in ulcerative colitis-associated colorectal dysplasias and cancers. *Cancer Res* **54**: 4841–4844

Terazono K, Yamamoto H, Takasawa S, Shiga K, Yonemura Y, Tochino Y and Okamoto H (1988) A novel gene activated in regenerating islets. *J Biol Chem* **263**: 2111–2114

Thibodeau SN, Bren G and Schaid D (1993) Microsatellite instability in cancer of the proximal colon. *Science* **260**: 816–819

Unno M, Yonekura H, Nakagawara K-i, Watanabe T, Miyashita H, Moriizumi S, Okamoto H, Itoh T and Teraoka H (1993) Structure, chromosomal localization, and expression of mouse *reg* genes, *reg*I and *reg*II. A novel type of *reg* gene, *reg*II exists in the mouse genome. *J Biol Chem* **268**: 15974–15982

Watanabe T, Yonekura H, Terazono K, Yamamoto H and Okamoto H (1990) Complete nucleotide sequence of human *reg* gene and its expression in normal and tumoral tissues: the *reg* protein, pancreatic stone protein and pancreatic thread protein are one and the same product of the gene. *J Biol Chem* **265**: 7432–7439

Watanabe T, Yonemura Y, Yonekura H, Terazono K and Okomato H (1994) Pancreatic beta cell replication and amelioration of surgical diabetes. *Proc Natl Acad Sci USA* **91**: 3589–3592

Zeng ZS, Sarkis AS, Zhang Z-F, Klimstra DS, Charytonowicz E, Guillem JG, Cordon-Cardo C and Cohen AM (1994) p53 nuclear overexpression: an independent predictor of survival in lymph node-positive colorectal cancer patients. *J Clin Oncol* **12**: 2043–2050

Zenilman ME, Perfetti R, Swinson K, Magnuson T and Shuldiner AR (1996) Pancreatic regeneration (*reg*) gene expression in a rat model of islet hyperplasia. *Surgery* **119**: 576–584

Zenilman ME, Magnuson TH, Perfetti R, Chen J and Shuldiner AR (1997) Pancreatic *reg* gene expression is inhibited during cellular differentiation. *Ann Surg* **225**: 327–332

*© 2000 Cancer Research Campaign*
